# Staging and haematological abnormalities of HIV-infected persons in the rural Free State Province of South Africa

**DOI:** 10.4102/phcfm.v3i1.222

**Published:** 2011-06-07

**Authors:** Andries J. Groenewald, Hendrik J. van Wyk, Corinna M. Walsh, Lynette J. van der Merwe, Sanet van Zyl

**Affiliations:** 1 Department of Chemical Pathology, University of the Free State, South Africa; 2 Department of Nutrition and Dietetics, University of the Free State, South Africa; 3 Department of Basic Medical Sciences, University of the Free State, South Africa

## Abstract

**Objectives:**

The objectives of this study were firstly to determine HIV (human immunodeficiency virus) prevalence in the rural Free State, secondly to classify the stages of HIV by utilising CD_4_ (cluster of differentiation 4) counts, and thirdly to measure differences in haematological abnormalities between HIV-uninfected and HIV-infected participants.

**Method:**

Blood specimens were obtained from 552 participants in Springfontein (36.3%), Trompsburg (30.1%) and Philippolis (33.5%). Participants were between 25–64 years of age, of which 28.1% were male (mean age 47.3 years) and 71.9% were female (mean age 46.0 years). The primary screening for HIV status was performed using the Enzygnost^®^ HIV Integral II Ag/ Ab test and confirmed by the Vironostica^®^ HIV Uni-Form II Ag/Ab test. Full blood counts were performed on all participants, but CD_4_ counts were only performed on HIV-positive serum.

**Results:**

The overall prevalence of HIV was 17.1%, with the peak prevalence in female participants (41.3%) occurring in the age group of 31–40 years, and in male participants (37.9%) in the age group of 41–50 years. Most HIV-uninfected participants (40.9%) were 51–60 years of age, whilst most HIV-infected participants were 31–40 years (35.6%) of age. The lowest mean CD_4_ count (276 cells/mm^3^) was observed in the age group 41–50 years, which was significantly lower than a mean count of 459 cells/mm^3^ in the age group 31–40 years (*p* ≤ 0.05). Haemoglobin was significantly reduced in HIV-infected male participants (*p* < 0.01) and female participants (*p* < 0.000 1), as ware white blood cell counts (*p* < 0.001), neutrofil counts (*p* < 0.005) and lymphocyte counts (*p* < 0.005). Peak prevalence of HIV in terms of age occurred later (between 31–40 years) than previously described and was reflected by a delayed low CD_4_ count (41–50 years).

**Conclusion:**

The low CD_4_ counts and anaemia were probably indicative of a generally ill study population. Participants in need of medical care should be identified and referred for management and follow-up.

## Introduction

The five countries with the highest HIV (human immunodeficiency virus) prevalence rates in the world are situated in Southern Africa and South Africa.^1^ The Nelson Mandela Trust and Human Science Research Council (HSRC) study of HIV and AIDS (Nelson Mandela Human Sciences Research Council 2002) was the first to describe the prevalence of HIV infection in the total South African population and to provide a detailed picture of the distribution and determinants of this devastating pandemic.

In a recent national community-based survey^3^ which included 7249 households and 13 518 individuals, the prevalence of HIV in the general population was 11.4%, with 12.8% prevalence in female participants and 9.5% prevalence in male participants. In formal urban areas, the prevalence in the Black population was 12.9%, 6.2% in the White population, 6.1% in the Coloured population, and 1.6% in the Indian population. Informal settlements in urban areas had a prevalence of 21.6%. The prevalence of HIV in urban formal and informal areas is higher (11.9% and 21.6%, respectively) than in rural formal and informal areas (7.8% and 8.8%, respectively). The peak prevalence occurred in women between ages 20–29 years (24.1%) and in men between 30–39 years (21.3%). The prevalence of HIV infection in the Free State province was reported to be 14.9%.^3^

In 2006, the prevalence of HIV in antenatal clinic attendees in South Africa was 29.1%, with the peak prevalence (38.7%) in the 25–29 years age group. In the Free State, 31.1% of attendees were HIV-infected, whilst KwaZulu-Natal had the highest prevalence at 39.1%.^4^

Staging is used to classify the disease into groups with different prognoses. Clinicians may use staging as a guide to treatment and medical management of patients. The proposed staging system for HIV infection constructed by the Centres for Disease Control and Prevention (CDC)^5^ has gained wide acceptance. The CDC uses CD_4_ counts of more than 500 cells/mm^3^, 200–499 cells/mm^3^ and fewer than 200 cells/mm^3^ to ‘guide clinical and therapeutic actions in the management of HIV-infected adolescents and adults’.^5^

A routine full blood count (FBC) can be part of either the general investigation of an acute illness, or regular monitoring of HIV infection, or of the side-effects of certain drug treatment regimens such as Zidovudine (AZT).^6^ Haematological abnormalities may give an indication of oxygen-carrying capacity as well as the risk of infections and bleeding tendencies.

This study formed part of the ‘Assuring Health for All in the Free State’ (AHA FS) research programme, a prospective epidemiological study with the main aim of determining how living in rural and urban areas affects a person's lifestyle and the indicators of health. A multidisciplinary research team investigated the sociodemographic status, the household food security, the dietary intake, the levels of physical activity, as well as the knowledge, attitudes and practices related to nutrition and reported the health status by using standardised questionnaires. In addition to a medical examination, anthropometric measurements and blood specimens were also obtained for various investigations. The rural baseline study was completed in 2007, whilst the urban baseline study was conducted in the Mangaung municipal district, which includes Bloemfontein, during 2009. The aim of this particular component of the study was to determine the prevalence of HIV infection in the rural Southern Free State, stage HIV-infected participants into different CD_4_ count categories, and measure the extent of haematological abnormalities in all participants.

## Ethical considerations

Approval to conduct the investigation was obtained from the Ethics Committee of the Faculty of Health Science at the University of the Free State (ETOVS nr: 21/7), the Department of Health and local municipalities. Before the onset of the study, all households were visited by trained fieldworkers and informed written consent to participate was obtained in the language of choice. Participation was voluntary and participants could withdraw at any time.

## Methods

The rural baseline study was performed over a 10-day period in March 2007. Fasting venous blood samples were obtained from 499 Black and Coloured households (*n* = 552) from Springfontein (36.3%), Trompsburg (30.1%) and Philippolis (33.5%) that are all situated more than 100 km from urban Mangaung. All volunteers between the ages of 25 and 64 years were eligible to participate. With the exception of age, no other exclusion criteria were applied.

Participation was voluntary. Trained fieldworkers were appointed in each community to visit all households in the Black and Coloured townships to explain the purpose of the study. Final-year and postgraduate students (from the Departments of Human Nutrition, Nursing and Social Work) conducted the interviews to complete household sociodemographic and individual health questionnaires under supervision of lecturers as part of the Service Learning functions of the University.

Medical examinations were performed at community clinics or halls by medical doctors from the Department of Basic Medical Sciences of the Faculty of Health Sciences at the University of the Free State, Bloemfontein. Limited medical service delivery in the region implies that it is possible that participants with a specific medical problem were more likely to participate in the study whilst bedridden participants may have been unable to visit the research venue to participate. More women than men took part in the study, most probably because more men are employed as labourers in the vicinity. As a result of the aforementioned reasons, we acknowledge that the study group is not in essence representative of the general population.

All information was treated as strictly confidential. Preintervention and postintervention counselling was given by trained medical practitioners. After completion of the rural baseline study, the participants were referred to the relevant local or provincial medical services for management and follow-up.

All participants were screened for their HIV status, using two fourth-generation serum assays. Primary screening for HIV status was performed using the Enzygnost^®^ HIV Integral II Ag/Ab test and confirmed by the Vironostica^®^ HIV Uni-Form II Ag/Ab test.

Serum CD_4_ counts were measured with the Beckman Coulter Epics XL^®^ flow cytometer. Full blood counts were obtained from blood collected in EDTA-containing tubes, using the Roche Sysmex XT 2000i^®^ analyser.

Descriptive statistics were used to analyse data. Variance statistics (*p* ≤ 0.05) were used to measure the significance of differences between results.

## Results

Participants (*n* = 552) aged between 25 and 64 years were included in the study, of which 28.1% were male (mean age 47.3 years) and 71.9% were female (mean age 46.0 years).

The HIV status of all participants was unknown. The prevalence of HIV infection in this survey was 17.1% (16.5% in female participants and 18.2% in male participants). The peak prevalence of HIV in female participants was observed in the age group 31–40 years (41.3%), and in male participants in the age group 41–50 years (37.9%). Springfontein had the highest prevalence of HIV infection (18.2%) and Trompsburg the lowest (15.3%).

The distribution of CD_4_ counts, which were performed only on HIV-infected participants, showed that 30.4%, 42.4% and 27.2% of participants had CD_4_ counts of higher than 500 cells/mm^3^, between 200–499 cells/mm^3^, and less than 200 cells/mm^3^, respectively.

**FIGURE 1:**
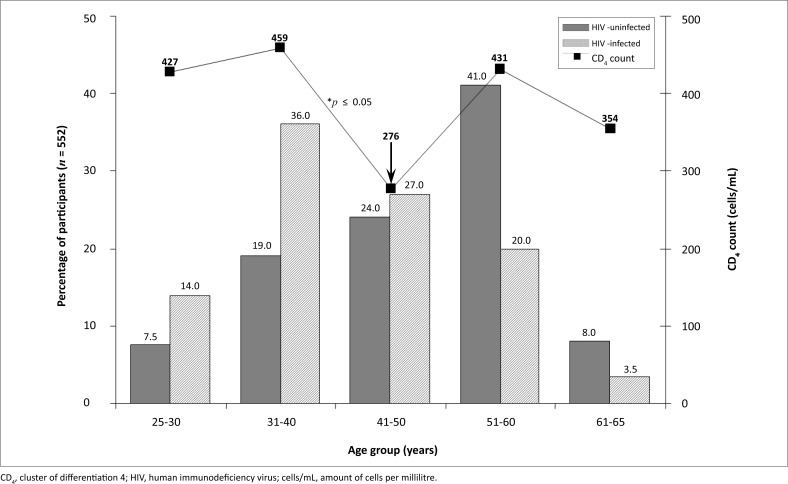
The distribution of HIV-infected participants and the mean CD_4_ counts of HIV-infected participants.

The age distribution of HIV-uninfected and HIV-infected participants as well as the distribution of mean CD_4_ counts in relation to age, are shown in Figure 1. Participants are grouped in 10 year intervals. The peak prevalence of HIV- uninfected and HIV-infected participants was at age 51–60 years (40.9%) and 31–40 years (35.6%), respectively. The lowest mean CD_4_ count (276 cells/mm^3^) was observed in the age group 41–50 years, which was significantly lower (*p* ≤ 0.05) than a mean CD_4_ count of 459 cells/mm^3^ observed in the age group 31–40 years.

The difference between the mean CD_4_ counts in the age groups 31–40 and 41–50 years (Figure 1) was statistically significant (*p* ≤ 0.05). The lowest mean CD_4_ count observed in the 41–50 years age group is indicated by the arrow in Figure 1.

Haematological results showed that HIV-infected participants had significantly reduced haemoglobin-values (for male participants *p* < 0.01, and for female participants *p* < 0.0001), neutrophil (*p* < 0.01) and lymphocyte values, in comparison to HIV-uninfected participants. No significant difference was observed between mean corpuscular volume and platelet counts of HIV-infected and HIV-uninfected participants (Table 1).

**TABLE 1:** Haematological findings in HIV-infected and HIV-uninfected participants.

Haematological components	Participants	HIV-uninfected		HIV-infected		*p*
	
		Mean	s.d.	Mean	s.d.	
Haemoglobin value (g/dL)	Male	15.3	1.3	14.4	1.72	< 0.01
	Female	13.9	1.28	13	1.34	< 0.000 1
White blood cell counts (x109/L)	-	7.4	2.24	6.5	2.6	< 0.001
Mean corpuscular volume (fL)	-	94.3	6.65	93.6	7.22	> 0.05
Platelet counts (x109/L)	-	278.6	74	269.2	72	> 0.05
Neutrophil counts (x109/L)	-	4	1.78	3.4	2.14	< 0.01
Lymphocyte counts (x109/L)	-	2.7	0.88	2.4	1.07	< 0.01

*Source*: Authors’ original data

HIV, human immunodeficiency virus; s.d., standard deviation; dL, decilitre (10^-1^ L); fL, femtoliter (10^-15^ L).

## Discussion

The Springfontein, Trompsburg, Philippolis area represents rural Southern Free State with mixed formal and informal settlements. The prevalence of HIV infection (17.1%) was slightly higher than previously reported (14.9%),^3^ which included urban settlements of the Free State and was therefore not comparable to our data. In South Africa, the prevalence of HIV was 7.8% in rural formal settlements and 8.8% in rural informal settlements,^3^ which was much lower than our observations. Our study group is probably not representative of the general population because of reasons previously outlined, and this may explain the higher, but not statistically significant (*p* ≤ 0.48) prevalence of HIV infection in Springfontein (18.2%) compared to Trompsburg (15.3%). It could also explain the prevalence differences between our results and published community-based surveys.^3^

It has been reported that HIV prevalence peaks in women aged 20–29 years (24.1%) and in men at the age of 30–39 years (21.3%).^3^ In our study, however, it was observed that HIV prevalence peaked later (between 31–40 years in women and 41–50 years in men). The observation that peak prevalence of HIV infection in women occurs at a younger age than in men, may reflect the fact that men tend to have sexual partners younger than themselves.^7^ The peak prevalence of HIV infection in our study occurred later than previously described,^3^ probably because our data, which included only rural participants, were compared to observations that represented participants from all over South Africa, including urban participants.

The CD4 count and the viral load test are essential parts of both the monitoring of the course of HIV infection over time, as well as the patient's response to treatment.^8^ CD_4_ counts of more than 500 cells/mm^3^ are associated with a healthy immune system, which weakens with progression of HIV infection until levels lower than 200 cells/mm^3^ are reached.^5^ Low CD_4_ counts are associated with a compromised immune system, serious infections and general health problems. In our study, the CD_4_ count was less than 200 cells/mm^3^ in 27.2% of HIV-infected participants, of whom none had received antiretroviral treatment. The peak prevalence of HIV infection in the age group 31–40 years supported the significantly low mean CD_4_ count (276 cells/mm^3^) in the age group 41–50 years (Figure 1). This finding suggests that it takes approximately 10 years from the onset of the infection with the virus until the progression into acquired immune deficiency syndrome (AIDS). It was previously observed that the median survival varied from 12.5 years for those aged 15–24 years at seroconversion to 7.9 years for those aged 45–54 years at seroconversion. The corresponding values for the development of AIDS were 11.0 years and 7.7 years.^9^

Haemoglobin was significantly reduced in HIV-infected male participants (*p* < 0.01) and female participants (*p* < 0.000 1) compared to uninfected participants (Table 1). Anaemia could have contributed to symptoms such as fatigue and breathlessness. It is more common amongst people with HIV infection and might be caused by HIV itself, opportunistic infections or the treatment.^6^ Significantly reduced haemoglobin values found in HIV-uninfected male participants (*p* < 0.01) and female participants (*p* < 0.0001) were probably indicative of a generally ill study population.

The number as well as the different types of white blood cells (Table 1) were measured. In contrast to HIV-uninfected participants, significantly reduced white blood cell counts (*p* < 0.001) and neutrophil counts (*p* < 0.005), might enhance the risk of bacterial and fungal infections in HIV-infected participants.^6^ The significantly reduced lymphocyte counts (*p* < 0.005) observed in our study are associated with HIV-related infection and killing of CD_4_ T-cells.^8^

It is recommended that the rural data obtained in this study be compared to that of an urban area to explain differences between our data and that of other studies. Data will be obtained from urban areas as well, as part of the continuing AHA FS study currently in progress in the Free State province.

## Conclusion

Low CD_4_ counts were associated with a compromised immune system, serious infections and general health problems. In our study, the CD_4_ count was less than 200 cells/mm^3^ in 27.2% of HIV-infected participants, of whom none had received antiretroviral treatment. Anaemia, also found in HIV-uninfected male participants and female participants, are probably indicative of a generally ill study population.

## Research significance

We determined the prevalence of HIV infection in the rural Southern Free State, staged HIV-infected participants into different CD_4_ count categories, and measured the extent of haematological abnormalities in all participants. Preintervention and postintervention counselling was provided by medical practitioners. After completion of the study, participants were referred to the relevant local or provincial medical services for management and follow-up procedures.
